# Genome-Wide Transcriptome Analysis Reveals that Cadmium Stress Signaling Controls the Expression of Genes in Drought Stress Signal Pathways in Rice

**DOI:** 10.1371/journal.pone.0096946

**Published:** 2014-05-09

**Authors:** Youko Oono, Takayuki Yazawa, Yoshihiro Kawahara, Hiroyuki Kanamori, Fuminori Kobayashi, Harumi Sasaki, Satomi Mori, Jianzhong Wu, Hirokazu Handa, Takeshi Itoh, Takashi Matsumoto

**Affiliations:** 1 Agrogenomics Research Center, National Institute of Agrobiological Sciences, Tsukuba, Ibaraki, Japan; 2 New Project Development Division, Hitachi Government & Public Corporation System Engineering, Ltd, Koto-ku, Tokyo, Japan; RIKEN Center for Sustainable Resource Science, Japan

## Abstract

Plant growth is severely affected by toxic concentrations of the non-essential heavy metal cadmium (Cd). Comprehensive transcriptome analysis by RNA-Seq following cadmium exposure is required to further understand plant responses to Cd and facilitate future systems-based analyses of the underlying regulatory networks. In this study, rice plants were hydroponically treated with 50 µM Cd for 24 hours and ∼60,000 expressed transcripts, including transcripts that could not be characterized by microarray-based approaches, were evaluated. Upregulation of various ROS-scavenging enzymes, chelators and metal transporters demonstrated the appropriate expression profiles to Cd exposure. Gene Ontology enrichment analysis of the responsive transcripts indicated the upregulation of many drought stress-related genes under Cd exposure. Further investigation into the expression of drought stress marker genes such as DREB suggested that expression of genes in several drought stress signal pathways was activated under Cd exposure. Furthermore, qRT-PCR analyses of randomly selected Cd-responsive metal transporter transcripts under various metal ion stresses suggested that the expression of Cd-responsive transcripts might be easily affected by other ions. Our transcriptome analysis demonstrated a new transcriptional network linking Cd and drought stresses in rice. Considering our data and that Cd is a non-essential metal, the network underlying Cd stress responses and tolerance, which plants have developed to adapt to other stresses, could help to acclimate to Cd exposure. Our examination of this transcriptional network provides useful information for further studies of the molecular mechanisms of plant adaptation to Cd exposure and the improvement of tolerance in crop species.

## Introduction

Heavy metal ions are highly reactive and toxic to living cells, and their accumulation in plants is a major agricultural problem. Heavy metals can be classified into two categories based on their influence on plant growth. The first category includes essential mineral elements, trace amounts of which may be required for adequate growth and development, and an excess of which is toxic as soon as the concentration exceeds the threshold that plants can endure. The second category includes non-essential metals with recognized toxicity including cadmium (Cd) and lead (Pb). In particular, Cd is absorbed by the roots from the soil and transported to the shoot, negatively affecting nutrient uptake and homeostasis in plants, even at low concentrations. It is also known to adversely impact various biochemical and physiological processes including changes in the transcriptome and proteome of plants, resulting in inhibited root and shoot growth and, ultimately, reduced yield [1], [2], [3]. Cd pollution in arable soil has dramatically increased worldwide over the last several decades through the use of phosphate fertilizers, sludge, and irrigation water containing Cd. Furthermore, accumulation of Cd in the edible parts of plants such as seed grains places humans at a risk when ingesting them because of its highly toxic effects on human health. Thus, it is important to study the mechanisms of plant responses and defenses to Cd exposure to overcome this problem.

Cd causes oxidative stress and generates reactive oxygen species (ROS) such as superoxide radicals (O_2_^−^) and hydrogen peroxide (H_2_O_2_), which can cause damage in various ways such as reacting with DNA causing mutation, modifying protein side chains and destroying phospholipids [4]. Many genes that respond to oxidative stress in plants, as well as genes associated with defense systems, have been studied, including induction detoxification enzymes such as glutathione S-transferase (GST), peroxidase (Prx), thioredoxins (Trx), peroxiredoxin (PrxR) and catalase (Cat) under Cd exposure, which confer Cd tolerance in plants [5], [6], [7], [8]. For defense against Cd toxicity, chelation of metal ions by ligands such as cysteine-rich metallothioneins (MT) and phytochelatins (PC) is also induced under Cd exposure. Such molecules bind Cd^2+^ ions through S-containing amino acid ligands and sequestrate the complexes to reduce cellular metal toxicity [1], [9], [10]. However, the manner in which the genes in these multigenic families respond to Cd has not been well investigated in rice.

Transporters with heavy metal binding domains are key factors for root uptake of Cd from soil and efflux pumping of Cd at the plasma membrane. The roles of transporters such as PDR9 (pleiotropic drug resistance-type ATP-binding protein 9) [11], LCT1 (low-affinity cation transporter 1) [12], and HMA3 (heavy metal ATPase3) [13] have been the focus of studies aimed at elucidating the mechanisms of Cd transport in rice. HMA3, which is located in the tonoplast, is well known to detoxify excess Cd by selectively sequestrating Cd into root vacuoles to decrease the concentration in the cell, so that translocation of Cd from the roots to the shoot is limited [13]. Cd transport is often caused by Fe or Zn transporters, such as IRT1 (iron-regulated transporter 1) and ZIP1 [Zrt (zinc-regulated transporter)/IRT-like protein 1], because of their low substrate specificity [14], [15], [16], [17]. HMA2 and MTP1 belong to the CDF (cation diffusion facilitator) protein family and are transporters of Zn and Cd [18]. Nramp5, a natural resistance-associated macrophage protein (NRAMP) family transporter, has been shown to transport Cd and Mn [19]. Although the functions of a few transporters for Cd transition and the effects of other ions on their expression have been reported in rice, a comprehensive overview of heavy metal transporters that change in expression under Cd exposure remains to be elucidated.

The recent elucidation of scaffolding mechanisms for Cd signaling pathways has begun to solve the puzzle of the complex Cd defense system in plants. Other signaling pathways contributing to the Cd stress response have not been well investigated but some important candidate genes, such as drought responsive element binding protein (DREB)/C-repeat binding factor (CBF), were identified to be involved by microarray-based approaches in rice [20]. However, a detailed view of the transcriptomic changes triggered by Cd exposure cannot be obtained with the Rice 44K Microarray (G2519F#15241, Agilent Technologies, Palo Alto, CA, USA) platform because it contains a probe set representing approximately 30,000 genes (the array probes were designed based on full-length cDNA structures), allowing for the detection of only 55% of the genes annotated in the rice genome [21].

Recently, the RNA-Seq strategy using next generation sequencers has become a useful tool for analyzing genome-wide gene expression to accurately quantify and catalogue all transcripts, including mRNAs and non-coding RNAs. With high resolution and sensitivity, RNA-Seq can provide detailed information on the transcriptional structure of genes [22]. The molecular mechanisms by which plants respond to changes in Cd stress are complex but of great importance, and could be useful in developing strategies for elucidating the gene networks involved in plant responses to various kinds of stress. Thus, we performed transcriptome analysis using RNA-Seq to compare gene expression profiles in seedlings of Cd-exposed and control (unexposed) rice plants (*Oryza sativa* L. cv. Nipponbare). We identified many novel responsive transcripts involved in signal transduction, antioxidation, detoxification and metal transport that might confer to tolerance to Cd exposure in rice. Moreover, we demonstrated that the overall gene expression of the Cd stress signaling network as an acute toxic response is involved in controlling drought stress signaling pathways by RNA-Seq analysis. This study and our previous study on Pi-stress [22] will contribute in understanding the genome-wide gene expression network of basal response to the stress in rice.

## Materials and Methods

### Sample preparation

Rice (*Oryza sativa* ssp. *japonica* cv. Nipponbare) seeds were germinated and grown by hydroponic culture in nutrient media [1.425 mM NH_4_NO_3_, 0.323 mM NaH_2_PO_4_, 0.513 mM K_2_SO_4_, 0.998 mM CaCl_2_, 1.643 mM MgSO_4_, 0.009 mM MnCl_2_, 0.075 mM (NH_4_)_6_ Mo_7_O_24_, 0.019 mM H_3_BO_3_, 0.155 mM CuSO_4_, 0.036 mM FeCl_3_, 0.070 mM citric acid, and 0.152 mM ZnSO_4_] [23] in a growth chamber at 28°C and 70–80% humidity in a 16h light/8h dark cycle. After 10 days, the seedlings of uniform size and growth were subjected to Cd stress treatment by transferring them to a similar medium with 50 µM CdSO_4_. The plants were maintained under Cd stress conditions for 120 h and then details of plant growth were recorded and sampling was performed as described previously [21]. Total RNA was extracted from all tissue samples using an RNeasy Plant Kit (Qiagen, Hilden, Germany) according to manufacturer's instructions. Construction of 13 cDNA libraries (2 tissues, 3 conditions, and 2–3 replicates) from total RNA using a TruSeq RNA sample preparation kit and sequencing with the Illumina Genome Analyzer IIx (Illumina Inc., San Diego, CA, USA) was performed according to the manufacturer's protocols.

### Sequencing and mapping of short reads onto the rice genome

More than two biological replicates for each set of conditions were highly correlated (coefficient > 0.92), and reads from the same treatment were merged for subsequent analysis. Trimming of Illumina adaptor sequences and low-quality bases (Q < 20) at the 5′ and 3′ ends of each read was performed by Cutadapt [24] (http://code.google.com/p/cutadapt/) and a custom-made program. These pre-processed reads were mapped to the IRGSP-1.0 genome assembly (http://rapdb.dna.affrc.go.jp/) to reconstruct the transcript structures by a series of programs; Bowtie for short-read mapping [25], TopHat for defining exon–intron junctions [26], and Cufflinks for gene structure predictions [27]. To estimate the expression levels of each transcript, all pre-processed reads were mapped to the Os-Nipponbare-Reference-IRGSP-1.0 genome assembly (http://rapdb.dna.affrc.go.jp/) by Bowtie with default parameters [28]. The expression level for each transcript was calculated as RPKM (Reads Per Kilobase exon Model per Million mapped reads)-derived read counts [29] based on the number of uniquely mapped reads that overlapped with exonic regions. The resulting RNA-Seq data were deposited in the DDBJ Sequence Read Archive (Accession No. DRA001092).

### Analysis of the responsive transcripts and alternative splicing patterns

We performed G-test to detect differentially expressed transcripts in control and Cd treatments based on the statistical null hypothesis that the proportions of mapped reads to the transcripts are the same between the two conditions. The frequency distribution of transcripts was determined by constructing 2×2 contingency tables with variables corresponding to the number of mapped and unmapped reads on a given transcript in control and each Cd treatment, respectively. A false discovery rate (FDR<0.01) was used in multiple hypothesis testing to correct for multiple comparisons. When calculating fold changes, 1 was added to avoid division by 0. Gene Ontology (GO) terms were assigned to each transcript from RAP-DB for each GO category. Enrichment of GO terms in the biological process category was evaluated by Fisher's exact test with a FDR threshold of 5% for responsive transcripts in major clusters at 1 h and 24 h after Cd treatment. The results were plotted as −log10 of FDR values in a heatmap. Microarray data (GSE6901, http://www.ncbi.nlm.nih.gov/geo/) were used to compare the responsive transcripts between Cd exposure (24 h) and other abiotic stresses (drought, salt and cold). The responsive transcripts (≥2-fold or ≤ 0.5-fold) in each treatment were used for Venn diagram analysis using the “venn” function of the R base package gplot version 2.10.1. For analysis of alternative splicing patterns in Cd-responsive genes, RPM (Reads per Million mapped reads) values of the splice sites in 5,222 representative loci were calculated using reads mapped on the sites. The splicing patterns in sites with RPM values > 1 were compared between control and Cd exposure samples.

### qRT-PCR

The expression of Cd-upregulated metal ion transporter genes in root and shoot samples were confirmed by quantitative RT-PCR analysis. Rice seeds were germinated and grown in water in a growth chamber. After 10 days, the seedlings were subjected to different stress treatments by transferring them to water containing different reagents (50 µM CdSO_4_ for Cd exposure, 50 µM AlCl_3_ for Al exposure, 50 µM CuSO_4_·5H_2_O for Cu exposure, 50 µM FeSO_4_·7H_2_O for Fe exposure condition, 50 µM HgCl for Hg exposure, 50 µM MgCl_2_·6H_2_O for Mg exposure, 50 µM MnCl_2_·4H_2_O for Mn exposure, 50 µM NaCl for Cl exposure, 50 µM NiCl_2_ for Ni exposure, 50 µM RbCl for Rb exposure or 50 µM ZnCl_2_ for Zn exposure). Ten-day-old rice seedlings transferred to water were used as a control for the stress treatment. Total RNA was extracted from samples collected after 24 h of each treatment. After DNase I (Takara, Shiga, Japan) treatment, first-strand cDNA was synthesized using the Transcriptor First Strand cDNA synthesis kit (Roche, Basel, Switzerland) according to the manufacturer's protocol. The resulting cDNA was used for PCR amplification in the LightCycler 480 system (Roche, Basel, Switzerland) with each primer set. The detection threshold cycle for each reaction was normalized using *Ubiquitin1* with 5′-CCAGGACAAGATGATCTGCC-3′ and 5′-AAGAAGCTGAAGCATCCAGC-3′ as primers. Three technical replicates for each treatment were used for analysis.

## Results and Discussion

### Changes in plant morphology under Cd exposure

**Figure 1:**
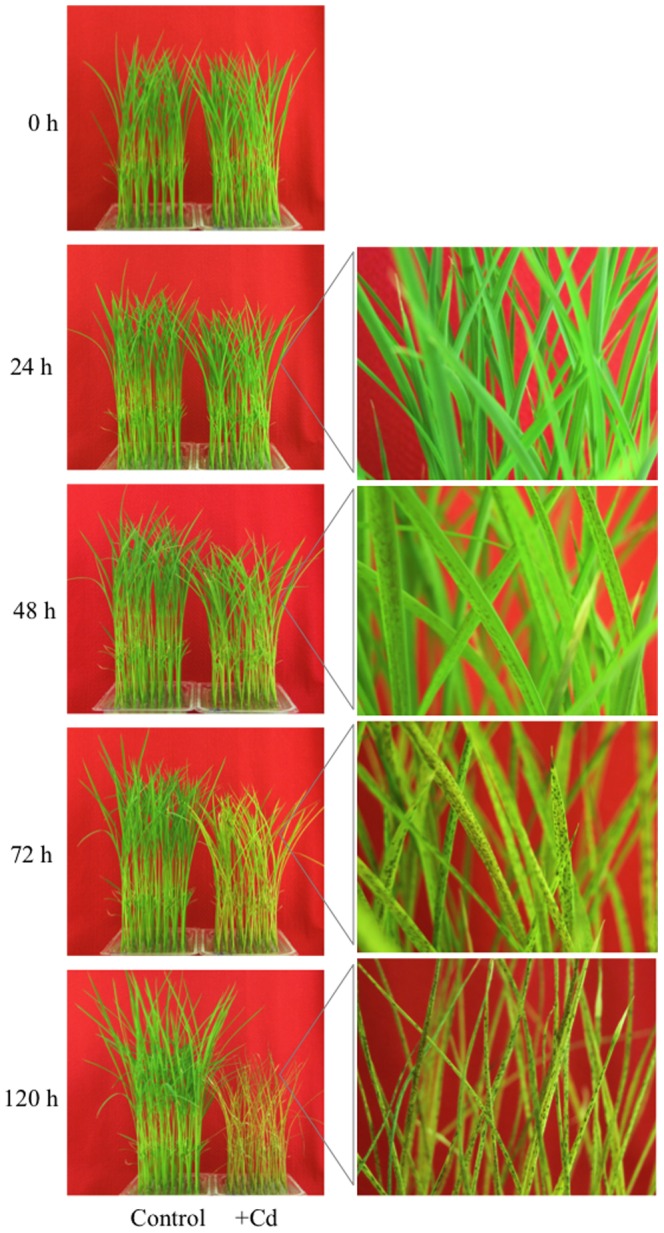
Rice phenotypes under Cd exposure. Phenotypic changes in rice plants grown from 0 to 120 h in culture medium with 50 µM CdSO_4_ for Cd stress. The shoots showed growth retardation. Black spots on the leaves gradually increased and leaves curled up slowly during Cd exposure. RPKM fold changes at 1 h and 24 h were calculated for Cd treated samples compared with non-treated samples (0 h).

In the hydroponically cultured rice, growth was greatly affected by Cd exposure (Figure 1). In Cd-exposed rice, growth retardation of the shoot was apparent after 24 h, many dark spots appeared on all leaves after 48 h, the leaves turned yellow and the leaf tips of the seedlings began to wilt after 72 h, and after 120 h of 50 µM cadmium exposure all leaf blades were curled completely and the seedlings were shrunken and wilting, which is attributable to supply of Cd (Figure 1). This concentration or higher concentrations of Cd have been previously shown to elicit robust physiological responses and gene expression as acute toxic responses in rice seedlings [3], [30], [31]. The wilting occurred gradually compared with drought treatment for 24 h, in which symptoms started to appear after 2 h treatment and plants were completely dried up after 24 h, in the same growth chamber (Figure S1). The detoxification processes of the plant are insufficient to cope with the toxic metal beyond a 10 µM dose, and wilting and reduction of plant fresh weight are accompanied by decreased leaf conductance and increased stomatal closing [32]. It has been suggested that part of the fatal damage to plants from Cd exposure occurs through drought stress.

### Cd treatment induced overall changes in gene expression in rice

**Figure 2:**
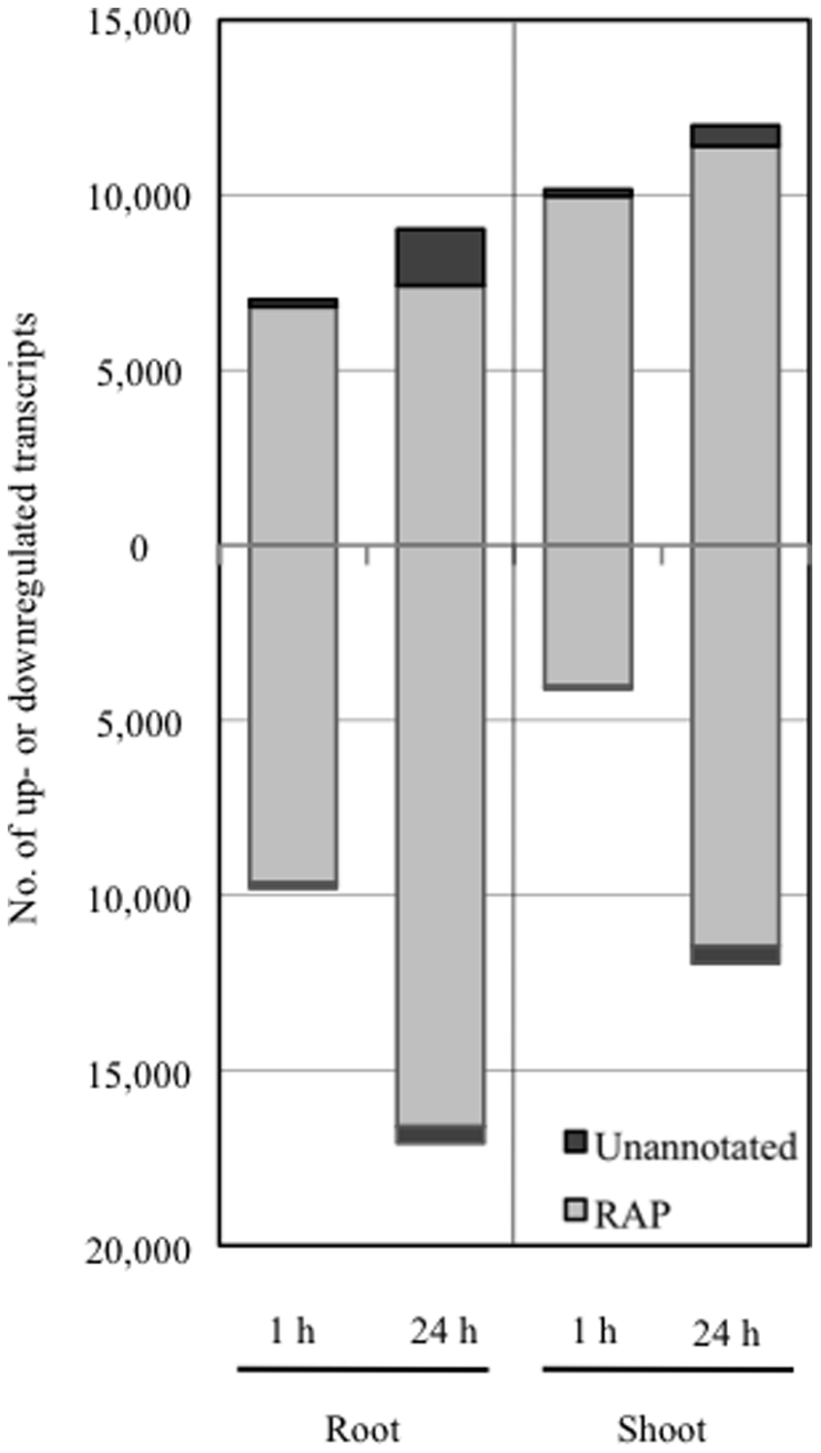
Distribution of upregulated and downregulated transcripts in roots and shoots responsive to Cd treatment. The total numbers of upregulated (upper) or downregulated (lower) transcripts in roots (left) and shoots (right) identified by RNA-Seq were determined by G-tests (FDR < 0.01) at each stress timepoint (1, 24 h) during Cd exposure in comparison with non-treatment (0 h). Each bar shows the distribution of transcripts with matching RAP-DB annotations (grey) and unannotated transcripts (black). The x-axis shows the time course and the y-axis shows number of transcripts.

To ascertain our hypothesis at the transcriptional level, we generated transcriptome profiles of the early response to Cd exposure using RNA-Seq during plant growth, particularly at 1 h and 24 h after 50 µM Cd treatment and before treatment (0 h). For each set of conditions, an average of approximately 48.3 million (90.7%) quality-evaluated reads were mapped to the rice genome sequence and used for further analysis (Table S1). Among the 60,163 transcripts detected in total (RPKM > 0), 36,222 were identified to be responsive to Cd exposure and were used to dissect the transcriptional responses associated with tissue (root and shoot) and time of collection (1 h and 24 h). The number of responsive transcripts was 16,814 in roots and 14,264 in shoots at 1 h, whereas it was 26,098 in roots and 23,924 in shoots at 24 h (Figure 2, Table S2), suggesting the effect of Cd exposure was enhanced gradually up to 24 h. Several *MT*s, *Prx*s and *heat shock proteins* (*Hsp*s) were strongly upregulated among the 20 genes with the greatest relative expression at 24 h. Table 1 shows strongly upregulated (top 20) transcripts without probes in the Rice 44K Microarray in roots and shoots at 24 h. We also found transcription factors (e.g. AP2-EREBP, NAC) that may function as regulatory factors under Cd exposure among these novel Cd-responsive transcripts, most of which had uncharacterized functions. Furthermore, the responsive transcripts included 1.84–7.98% unannotated transcripts predicted by the Cufflinks program in each particular tissue/time. A validation experiment on the responsive unannotated transcripts was performed by qRT-PCR analysis [21]. These novel transcripts may represent interesting novel targets to understand Cd regulatory pathways in rice. Moreover, among 44,519 representative loci on the rice genome (IRGSP-1.0), alternative splicing isoforms were confirmed in 5,222 (11.7%) representative loci. Changes in the alternative splicing patterns of these loci were examined against Cd exposure and 2,873 loci (6.5%) showed different patterns. RNA-Seq is a promising tool for analyzing alternative patterns that microarrays are unable to, and approximately ∼48% of rice genes showed alternative splicing patterns [33]. This suggests that Cd responsive expression is also regulated by alternative splicing mechanisms with respect to the timing of the response or tissue specificity. In conclusion, RNA-Seq data were far superior to data derived from the microarray and that Cd treatment affected many genes and caused drastic changes in gene expression in rice.

**Table 1:** Cadmium-upregulated transcripts identified in roots and shoots by RNA-Seq analysis.

Root 24 h	Description	Fold change
*Os12t0568166-01*	Conserved hypothetical protein	341.43
*Os05t0211700-00*	-	210.16
*Os08t0404900-00*	Conserved hypothetical protein	170.60
*Os09t0492900-00*	Conserved hypothetical protein	170.46
*Os01t0653300-00*	VQ domain containing protein	151.31
*Os01t0661750-00*	Conserved hypothetical protein	142.92
*Os06t0133500-00*	Conserved hypothetical protein	129.63
*Os06t0662550-01*	Conserved hypothetical protein	108.59
*Os07t0154201-00*	Hypothetical gene	103.63
*Os06t0147250-00*	Conserved hypothetical protein	102.14
*Os02t0464550-01*	Conserved hypothetical protein	101.50
*Os11t0495400-00*	PHF5-like protein	100.00
*Os04t0429050-00*	AP2-EREBP	97.98
*Os10t0525200-01*	Cytochrome P450 family protein	93.75
*Os01t0498802-01*	Non-protein coding transcript	93.09
*Os12t0418600-01*	Hypothetical conserved gene	82.22
*Os07t0162000-00*	-	82.14
*Os06t0146650-00*	Conserved hypothetical protein	70.88
*Os08t0336200-01*	Hypothetical gene	70.61
*Os10t0525301-00*	Hypothetical gene	68.00

Shoot 24 h	Description	Fold change
*Os03t0291500-01*	Asparagine synthetase	1179.82
*Os03t0797500-01*	Indole-3-glycerol phosphate lyase	543.10
*Os03t0797551-00*	-	314.67
*Os04t0486950-01*	Malate synthase	218.28
*Os03t0277650-00*	-	149.55
*Os07t0147550-00*	Photosystem II 10 kDa polypeptide	119.54
*Os11t0117400-00*	WRKY	93.44
*Os12t0123700-00*	NAC	64.07
*Os02t0121750-00*	Hypothetical protein	63.74
*Os12t0512150-00*	Hypothetical protein	60.14
*Os04t0108101-00*	Hypothetical protein	56.30
*Os02t0783650-01*	Hypothetical gene	55.23
*Os02t0829850-00*	Conserved hypothetical protein	53.62
*Os05t0460000-01*	HSP70	52.63
*Os12t0191601-00*	Hypothetical protein	49.63
*Os02t0106201-00*	Hypothetical protein	46.6
*Os10t0379233-00*	Hypothetical protein	41.77
*Os04t0180500-00*	Hypothetical protein	41.5
*Os12t0639500-01*	ATPase, AAA-type, core domain containing protein	39.69
*Os01t0609501-00*	Hypothetical protein	39.49

Reads were mapped to the rice genome and responsive genes were identified by G-tests. The top 20 upregulated transcripts in roots and shoots that are not represented on the Rice 44K microarray platform are shown.

### Functional characterization of Cd-responsive transcripts

To investigate the functions of cadmium stress-responsive transcripts, we performed Gene Ontology (GO) analysis and found an overrepresentation of specific GO keywords that denote involvement with processes triggered by stress, including metal ion transport (GO:0030001) (upregulated, root), response to stress (GO:0006950) (upregulated, root and shoot), trehalose biosynthetic process (GO:0005992) (upregulated, shoot), DNA replication (GO:0006260) (downregulated, root), DNA repair (GO:0006281) (downregulated, root), translation (GO:0006412) (downregulated, root and shoot), and photosynthesis (GO:00015979) (downregulated, shoot) (Figure S2). These enriched GO terms among the upregulated transcripts indicate that RNA-Seq was successful in identifying Cd-responsive genes. The enriched GO terms among downregulated transcripts result in growth retardation (Figure 1). These results imply that Cd controls a significant part of the defense to stress and plant growth.

Among the GO keywords identified, response to stress (GO:0006950) included many drought stress responsive genes such as the *Arabidopsis* cor47 homolog *Dip1* [34], *Rab 21* ( =  *Rab16A*) (responsive to abscisic acid 21) genes [35], and other *Rab 16 genes* [36] in roots and shoots at 24 h under Cd exposure (Table S3). The signaling pathways in drought, high-salinity and low temperature stress show high levels of cross-talk among abiotic stresses in *Arabidopsis* [37]. Our results suggested that expression of these genes responsive to drought stress (also implying a relationship to high-salinity and low temperature stresses) was affected by Cd exposure, but a relationship between Cd and drought stresses at the transcription level has not been reported as far as we know.

### Cd exposure triggered upregulation of drought stress responsive genes

We investigated the expression of well-characterized drought stress-related transcription factors (TFs) and their downstream genes under Cd exposure to ascertain the relationship between Cd and drought stresses.

#### i) DREB/CBF TFs

**Figure 3:**
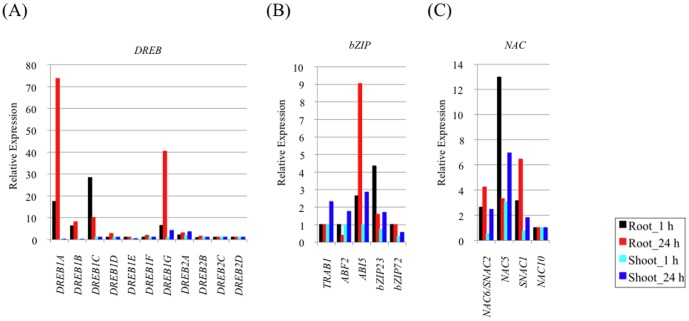
Effects of Cd exposure on rice growth and the expression of abiotic stress-related TF genes. The relative expression of (A) *DREBs*, (B) *bZIPs* and (C) *NACs* in rice is shown. The x-axis shows transcripts and the y-axis shows relative expression. The black bar shows the relative expression in roots at 1 h, the red bar roots at 24 h, the light blue bar shoots at 1 h and the blue bar shoots at 24 h.

Among the *DREB/CBF* TFs, which contain drought-responsive elements (DRE), *DREB1A/CBF3* and *DREB1G* were drastically upregulated in roots after 24 h of Cd exposure and *DREB1C/CBF2* was drastically upregulated in roots after 1 h (Figure 3A). Over-expression of *DREB1A/CBF3* improved tolerance to drought, high-salinity and low temperature, and resulted in growth retardation under non-stress conditions, suggesting functional similarity to *Arabidopsis* [38], [39]. Over-expression of *DREB1G* significantly elevated tolerance to drought [40]. As the transcriptional networks mediated by DREB1 are conserved in plants and DREB1 regulates many downstream target genes under abiotic stress [37], part of the signaling pathway related to DREB1 might be enhanced under Cd exposure in rice.

#### ii) bZIP TFs

Several bZIP domain TFs were characterized that regulated expression of ABA-responsive genes in rice. The abiotic stress-inducible genes *bZIP23* [41] and *ABI5* [42] were upregulated in roots at 1 h and 24 h during Cd exposure, respectively (Figure 3B). *bZIP23* and *ABI5* are thought to function as key components in ABA-dependent transcriptional networks in rice, suggesting the existence of gene expression regulation through ABA-dependent pathways under Cd exposure. The existence of an ABA-dependent pathway was supported by strong upregulation of *NCED3* and *NCED9* in roots (Table S3). In our BLASTP search, the *Arabidopsis* proteins AtNCED3 [43] and AtNCED9 [44], key enzymes of ABA synthesis during drought stress and seed development, respectively, were the top hits to NCED3 and NCED9 in rice. ABA is known to accumulate rapidly and confer stress tolerance by inducing many genes under drought [43], [44], [45], [46]. Synthesis of ABA following Cd stress can lead to decreased leaf conductance as Cd^2+^ toxicity perturbs the plant–water relationship [32] (Figure 1). *LEA3-1* was upregulated in *bZIP23* overexpressors [41], and here it was also upregulated in roots and shoots (Table S3). *LEA* genes often appear to be ABA-dependent [47], and their proteins may function in protecting macromolecules such as enzymes, protein complexes and membranes [48]. Overexpression of *LEA3-1* in rice can significantly improve relative yield under drought stress [49].

#### iii) NAC TFs

Several NAC TFs, including *NAC5* [50] and *SNAC1* [51], are induced by drought, high-salinity and low-temperature. *NAC5* was upregulated in roots at 1 h and shoots at 24 h, and *SNAC1* was upregulated in roots at 24 h during Cd exposure (Figure 3C). *NAC5* is regulated through an ABA-dependent pathway [52]. *LEA3*, which was identified as a *NAC5* downstream gene [50], [53], and a rice ERD1 (early responsive to drought 1) homologue (*OsERD1*) were also upregulated in roots and shoots (Table S3). The expression of *OsERD1* is probably regulated by SNAC1 through the NAC recognized sequence (NACRS) promoter [51], [54]. Over-expression of *SNAC1* can significantly improve drought resistance and confer strong tolerance to high-salinity stress [51].

#### iv) Other TFs

Many other stress-related TFs, including AP2/ERFs (*AP37*, *AP59*), C2H2 zinc finger (*ZFP252*), TIFY (*TIFY11*) and MYB (*Myb4*), were also upregulated under Cd exposure (Table S3). Overexpression of *AP37*, *AP59* [55] and *ZFP252* [56] results in drought and high-salinity tolerance. Overexpression of *ZFP252* in rice increased the amount of soluble sugars and free proline [56]. The accumulation of soluble raffinose and the upregulation of *galactinol synthase* genes, key enzymes of raffinose synthesis, have been reported in drought-, high salinity- and low temperature-treated *Arabidopsis* plants [57]. In our study, *galactinol synthase* and *raffinose synthase* genes were strongly upregulated in roots under Cd exposure (Table S3). Cd treatment has also been shown to increase the level of raffinose in *Arabidopsis* [58]. The *AtGolS3* galactinol synthase gene is controlled by DREB1A through DRE and DRE-like *cis*-elements in *Arabidopsis* [57], [59]. These results suggest raffinose is a metabolite for adaptation to Cd and might be produced by enhancing a DREB-related pathway in rice. *P5CS*, a key enzyme of osmoprotectant proline synthesis, was upregulated in shoots under Cd exposure (Table S3). Accumulation of proline as an enzyme protectant has been reported in rice seedlings exposed to Cd [60], [61], suggesting that proline is also a metabolite for adaptation to Cd. Overexpression of *TIFY11a* [62] resulted in high-salinity and drought tolerance, and overexpression of *Myb4* [63] resulted in tolerance to low temperature, so upregulation of *TIFY11* and *Myb4* may also function in tolerance to Cd exposure (Table S3). Thus, many reports suggest that overexpression of stress-inducible TFs can increase abiotic stress tolerance to drought, high-salinity, or low temperature in plants, so upregulation of such TFs could be useful for developing transgenic crops with enhanced tolerance to Cd stress.

Some (*DREB*, *bZIP*s and *NCED*) were more upregulated in roots than in shoots under Cd exposure up to 24 h and the tendency of their expression patterns was confirmed by qRT-PCR analysis, which was different to the tendency under drought stress for 3 h (Figure S3, Table S4). As *OsDREB1A* and *OsDREB1B* were included in the 50 genes with the greatest relative expression in roots exposed to 10 µM Cd-treatment for 3 h [20], roots might be affected more directly and earlier by Cd exposure. In conclusion, our data clearly indicated that Cd affects the expression of genes in the drought-related signaling pathway, especially in roots, because the Cd exposure treatment was performed in hydroponic culture.

We also confirmed the upregulation of Jacalin1, LOX (lipoxygenase), BBTI 1 (Bowman Birk trypsin inhibitor 1), Receptor kinase containing LRR repeats, PSLS (Phospho sulfolactate synthase), Hsp70, PP2Ca and PP2Cb in one or more of the tissue/treatment combinations (Table S3). The upregulation of these genes was ascertained to help plants acclimate to stress conditions, such as drought in AtCBF3- or AtABF3-overexpressing transgenic rice [64]. These results suggest that Cd exposure more or less perturbs the expression of genes in drought, high salinity- and low-temperature stress signaling pathways, which results in similar morphological changes in response to both Cd and drought (Figure 1, Figure S1). Even though comparative analysis using microarray data revealed that 28.0–54.3% of the responsive transcripts (≥2-fold or ≤ 0.5-fold) responded to other abiotic stresses (drought, high salinity or low-temperature) in roots and shoots (Figure S4), Cd-responsive transcripts responded to drought/high-salinity stresses more than twice as much as to low temperature stress (Figure S4), suggesting there are higher levels of cross-talk between Cd and drought/high-salinity stresses than in the cross-talk between Cd and low temperature stress as expected from observation of wilted seedlings under Cd exposure (Figure 1). We also identified that 9% of Cd-upregulated transcripts were commonly upregulated among the four stresses, including SNAC1, ZEP252 and Myb4 (Table S5), which may confer general adaptation under abiotic stress.

### Upregulation of genes in the defense system to Cd exposure and RNA-Seq revealed the regulated genes of multigenic families

Next, we investigated the expression of antioxidant and detoxification enzymes to confirm that our expression profiles reflected the response to Cd exposure.

#### i) Antioxidant enzymes for ROS-scavenging

**Figure 4:**
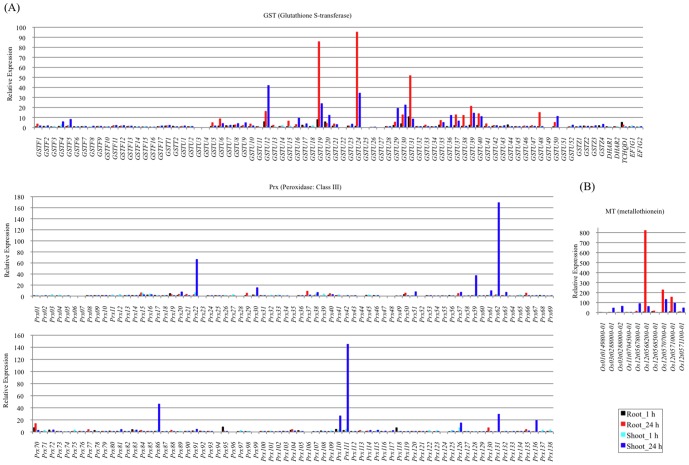
Relative expression of transcripts that may function in defense and detoxification under Cd treatment. RPKM fold changes at 1 h and 24 h were calculated for Cd treated samples compared with non-treated samples (0 h). (A) Relative expression of the *GST* gene family, (B) the *Prx* gene family, and (C) the *MT* gene family are shown. The x-axis shows transcripts and y-axis shows relative expression. The black bar shows the relative expression in roots at 1 h, the red bar roots at 24 h, the light blue bar shoots at 1 h and the blue bar shoots at 24 h.

The production of ROS is unavoidable under various biotic and abiotic stresses including Cd exposure. The accumulation of ROS is largely counteracted by an intricate antioxidant defense system [6]. The enzymatic scavengers GST [65] (Pfam accession: PF02798.15, PF13417.1, PF13410.1, PF00043.20, http://pfam.sanger.ac.uk/) and class III Peroxidase (Prx) (E.C. 1.11.1.7) [66] have large and complex gene families that control key metabolic steps in many eukaryotic systems. Among 80 GST genes, the responsive genes tended to be more upregulated in roots at 24 h compared with the other tissue/treatment combinations. In particular, *GSTU12* (shoot), *GSTU19* (root and shoot), *GSTU24* (root and shoot), *GSTU30* (shoot), *GSTU31* (root) and *GSTU39* (root) were strongly upregulated [fold change (FC) > 20] (Figure 4A). *GSTU19*, *GSTU24* and *GSTU39* were found to be upregulated in the roots of rice seedlings under arsenate exposure using a microarray platform [67], suggesting that part of the Cd defense might be similar to the As defense in the roots of rice. Enhanced GST with glutathione peroxidase activity in transgenic tobacco increased glutathione-dependent peroxide scavenging and alterations in glutathione and ascorbate metabolism that led to reduced oxidative damage [68]. Class III Prx genes catalyze the reduction of H_2_O_2_ by transferring electrons to various donor molecules such as lignin precursors and secondary metabolites [69], [70]. *Prx22*, *Prx59*, *Prx62*, *Prx86*, *Prx110*, *Prx111* and *Prx131* among 138 *Prxs* were upregulated strongly (FC > 20) in shoots after 24 h of Cd exposure (Figure 4A). *Prx86 and Prx111* were strongly upregulated in the plant defense against a gall midge [71] and against *Xanthomonas oryzae* and *Magnaporthe grisea* [72]. As many studies have described the diverse functions of Prx in *Arabidopsis* [8], [73], these genes might be expressed in a time and tissue specific manner in rice.

Genes encoding various kinds of ROS-scavenging enzymes, such as glutaredoxin (Grx) [74], Trx [75], PrxR [76], monodehydroascorbate reductase (MDAR) [77], alternative oxidase (AOX) [78], Cat (PF00199), and alpha-dioxygenase (*α*-DOX1) [79] were also upregulated (FC > 3) (Figure S5), suggesting activation of several antioxidative systems in the plant cells under Cd exposure. Most of these genes tended to be upregulated more at 24 h compared with 1 h after Cd exposure. α-DOX1, which plays a role in protecting tissues from oxidative damage [80], was the most upregulated (∼94-fold) in shoots at 24 h under Cd exposure. *α-DOX1* is well-known to be upregulated and show increased activity under oxidative- and heavy metal-stresses [80]. Tissue-specific upregulation was observed for some genes, such as *Os01g0194600* (*Grx*) in roots and *α-DOX1* in shoots. Even though the genes were from the same family, we observed tissue-specific expression for different *PrxR* and *AOX* genes. One of the two genes in each family was upregulated more in roots compared with shoots and the other gene showed an opposite upregulation pattern (Figure S5). Respiratory burst oxidase homolog (Rboh: NADPH oxidase) genes that produce ROS [6], [81] were also upregulated (Figure S5). These results indicate that the treatment evokes ROS in rice cells. However, while the gene expression profiles of the whole multigenic family have not been well investigated under Cd exposure, the regulated genes in the multigenic family were elucidated clearly by RNA-Seq analysis. Through the use of gene transfer technology to manipulate the activities of these antioxidant enzymes, ROS-scavenging systems in plants might be used to increase plant stress tolerance.

#### ii) Detoxification enzyme through chelation

The chelation of heavy metals by particular ligands is a well-characterized mechanism of Cd detoxification that has evolved in plants. The two best-characterized heavy metal-binding ligands in plant cells are PC and MT, which are involved in detoxification [9]. PC is synthesized from reduced glutathione (GSH) in a transpeptidation reaction [82]. PC synthase (PF05023.9) and GSH synthase (PF04107, PF03917, PF03199) genes did not show much response to Cd exposure in this study, but *cad1*, a PC deficiency mutant in *Arabidopsis*, is Cd sensitive [83]. MTs have been found in diverse organisms including mammals, plants, and fungi as well as some prokaryotes and function in maintaining homeostasis of essential metals and metal detoxification [1], [9]. MTs are rich in cysteine residues that coordinate multiple metal atoms such as cadmium, zinc and copper. Eleven MT genes were found to be differentially expressed during growth and development, in various tissues and during biotic and abiotic stresses [84], [85]. Here, 9 of these 11 MT genes were strongly or weakly upregulated in at least one set of conditions (Figure 4B). Interestingly, two sets of three MT genes (*Os12g0567800*, *Os12g0568200*, *Os12g0568500* and *Os12g0570700*, *Os12g0571000*, *Os12g0571100*) reside on chromosome 12 within 40-kb and 15-kb regions, respectively. As the clustering of MTs is well-known in mouse and human [86], these might have evolved from gene duplications in rice. These clustered genes were upregulated at different levels in at least two of the tissue/treatment combinations (Figure 4B). *Os12g0568200* showed the strongest upregulation (821-fold) among them, in roots at 24 h under Cd exposure (Figure 4B). Simultaneously, *cis*-regulatory changes and minor changes in the regulatory regions of genes may occur during evolution [87], [88], resulting in diversity of gene expression levels. Members of the rice MT gene clusters differed in their tissue expression patterns, suggesting that each gene may perform different functions in specific tissues. The responsive transcripts of antioxidative and detoxification enzymes differed in their expression patterns among metal stresses (Figure S6, Table S4). Their upregulation indicated that our expression profiles under Cd exposure obtained by RNA-Seq analysis were reliable.

### Responses of various metal transporter genes under Cd and other metal stresses

To confirm whether other ions affect the expression of Cd-responsive genes, we investigated the expression of metal transporter genes from the HMA, MatE (multi antimicrobial extrusion protein) [89], Zip, CDF, NRAMP, and PDR families, as well as LCT1 [12] and LCT1 homologues (*Os06g0576200*, *Os06g0674000*), because at least one gene from each of these families and LCT1 have been reported to function in Cd transport. As LCT1 did not have any Pfam domain for heavy metal binding, we searched for homologues in RAP DB using a BLASTP search (threshold of 1e-03). These gene families contain specific metal ion binding domains [PF01554 (MatE), PF08370 (PDR_assoc), PF01545 (Cation_efflux), PF02535 (Zip), PF00403 (HMA), PF01566 (Nramp)], which may function in Cd transport. In total, 88.9% of the transporters (168 transcripts) were responsive to Cd exposure, with 35.7% (60 transcripts) in the HMA family, 29.8% (50 transcripts) in MatE, 10.7% (18 transcripts) in Zip, 7.7% (13 transcripts) in Cation_efflux and Nramp, 6.0% (10 transcripts) in PDR_assoc and 2.4% (4 transcripts) being LCT1 and LCT1 homologue. *PDR9* [11], *LCT1* [12] and *HMA3* [13], which are important of Cd translocation in rice, were identified as the responsive transcripts. Transcripts upregulated more than 5-fold in one or more of the tissue/treatment combinations are shown in Table 2. *Os02t0585200* containing an HMA domain was the most upregulated in roots (87-fold), and *Os01t0609900* containing a PDR_assoc domain was the most upregulated in shoots (53-fold) at 24 h during Cd exposure. These results emphasized the potential of the RNA-Seq strategy to reveal novel Cd-responsive transporter transcripts in rice. It should be mentioned that the expression levels were diverse among the gene families, suggesting they may have differentiated functions in transporting various metal ions.

**Table 2:** Cadmium-responsive metal ion transporters identified in rice.

		Root		Shoot	
Family	Transcript	1 h	24 h	1 h	24 h
HMA	*Os02t0585200-01*	**10.78**	**87.00**	0.27	2.11
	*Os03t0152000-01*	**7.68**	**34.42**	0.09	0.91
	*Os02t0584800-01*	3.15	**33.27**	0.15	0.78
	*Os02t0585100-00*	**5.67**	**23.19**	1.03	**5.24**
	*Os02t0584700-01*	3.32	**17.49**	0.23	1.09
	*Os03t0372600-00*	2.48	**9.90**	1.21	2.41
	*Os02t0530100-02*	0.98	**7.17**	0.55	0.42
	*Os02t0530100-01*	0.92	**7.03**	0.56	0.45
	*Os01t0976300-01*	3.07	**6.36**	0.96	**13.77**
	*Os04t0244800-01*	**5.67**	3.67	1.95	**5.61**
	*Os06t0542300-01*	1.39	2.35	0.62	**15.21**
	*Os06t0665800-01*	1.06	2.33	1.51	**5.90**
	*Os08t0403300-00*	0.49	1.73	1.07	**6.41**
	*Os03t0178100-00*	1.10	0.67	1.85	**5.25**
MatE	*Os10t0344000-01*	1.05	**12.19**	0.63	**13.65**
	*Os03t0188100-01*	1.75	**9.77**	1.22	**14.48**
	*Os10t0345100-01*	2.63	4.53	2.04	**6.69**
	*Os04t0571600-01*	**10.32**	2.55	1.13	0.25
	*Os03t0572900-01*	1.18	0.97	1.45	**7.38**
	*Os07t0502200-01*	2.45	1.30	2.65	**6.24**
	*Os01t0504500-02*	0.97	0.90	1.10	**5.39**
	*Os02t0676400-00*	**7.77**	0.73	1.12	1.13
Zip	*Os03t0411800-01*	1.21	**10.56**	1.20	**14.60**
	*Os01t0972200-00*	**5.06**	1.69	1.39	2.07
Cation_efflux	*Os01t0130000-01*	1.45	1.02	1.46	**6.70**
	*Os01t0130000-02*	1.28	0.92	1.30	**5.82**
Nramp	*Os07t0258400-02*	0.66	**5.05**	1.08	1.50
PDR_assoc	*Os01t0342750-01*	0.75	**5.82**	0.91	2.47
	*CUFF.28142.2*	2.97	2.43	2.47	**45.91**
	*Os01t0609900-02*	1.75	2.14	1.77	**53.64**
	*Os01t0609300-01*	1.55	2.03	1.03	**32.64**
	*Os08t0384500-01*	1.51	1.30	1.59	**5.20**
	*Os01t0609200-00*	**6.67**	0.74	1.04	1.15
LCT1	*CUFF.25087.1*	1.11	1.57	1.87	**5.65**
	*CUFF.25087.2*	1.08	1.48	1.96	**6.04**
	*CUFF.25087.3*	1.12	1.49	1.95	**5.97**

Metal ion transporters containing Pfam domains [PF01554 (MatE), PF08370 (PDR_assoc), PF01545 (Cation_efflux), PF02535 (Zip), PF00403 (HMA), PF01566 (Nramp)], LCT1 (unannotated transcripts by RAP, identified by the Cufflinks program) and LCT1 homologues upregulated more than 5-fold in one or more of the tissue/treatments combinations are shown. Bold characters show fold changes greater than 5 in upregulated transcripts. *CUFF.28142.2* (chr07: 20207865..20213557) *CUFF.25087.1* (chr06: 22566131..22572032), *CUFF.25087.2* (chr06: 22566593..22572032) and *CUFF.25087.3* (chr06: 22567729..22571545) were identified by the Cufflinks program.

**Figure 5:**
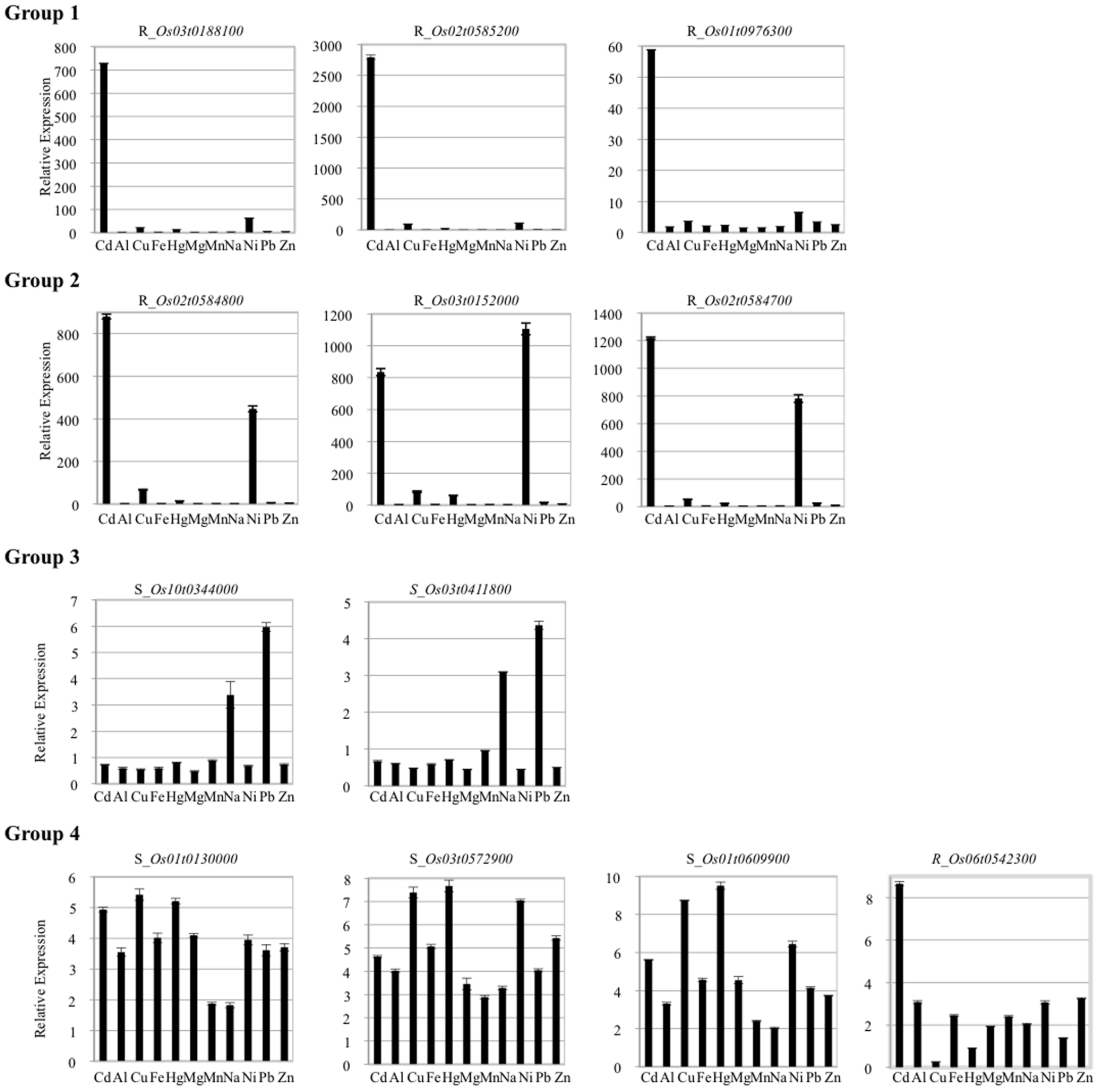
Expression patterns of Cd-upregulated heavy metal ion transporters in various medium conditions by qRT-PCR analysis. The expression of Cd-upregulated heavy metal ion transporters was investigated in various liquid media containing different kinds of metal ions by qRT-PCR analysis. The x-axis shows treatments and the y-axis shows relative expression. Transcript expression levels were normalized using an internal control (*ubiquitin 1*) and plotted relative to expression in water (control) at 24 h in roots (*R*) and shoots (*S*). The transcripts were classified into four groups based on their expression patterns.

Next, the expression of 12 randomly selected upregulated transporter genes was analyzed by qRT-PCR in seedlings treated with various metals, such as Zn, Mg and Pb (Figure 5, Table S4). The seedlings were grown in water for 10 days to avoid priming effects before transferring to various media. The expression patterns of the Cd-upregulated transporter transcripts were classified into four groups: three transcripts (MatE, HMAs) upregulated significantly under Cd exposure (group 1), three transcripts (HMAs) upregulated significantly under both Cd and the other treatments (group 2), two transcripts (MatE, Zip) upregulated under the other treatments but not Cd (group 3), and four transcripts (Cation efflux, MatE, PDR_assoc, HMA) upregulated under general stress (group 4), though their expression might have been affected by ion concentration, treatment time, the balance of other ions, tissue and growth stage. The transcripts of group 1 might respond specifically to Cd. In our conditions, the transcripts of group 2 were not only upregulated by Cd, but also by the essential metal Ni, suggesting they might function in Ni transport. The nickel transporter TjZnt in the Ni hyperaccumulator *Thlaspi japonicum* has been reported to have Cd^2+^ transport ability [90]. The transcripts of group 3 did not respond to Cd in water substituted for nutrient-rich medium, suggesting they were easily affected by other metal ions. The transcripts of group 4 might respond to general stress. The expression of most of these was affected to different degrees by exposure to other metal ions, suggesting specific systems for transporting Cd may have not developed in rice because Cd is a non-essential metal for the plant.

## Conclusions

**Figure 6:**
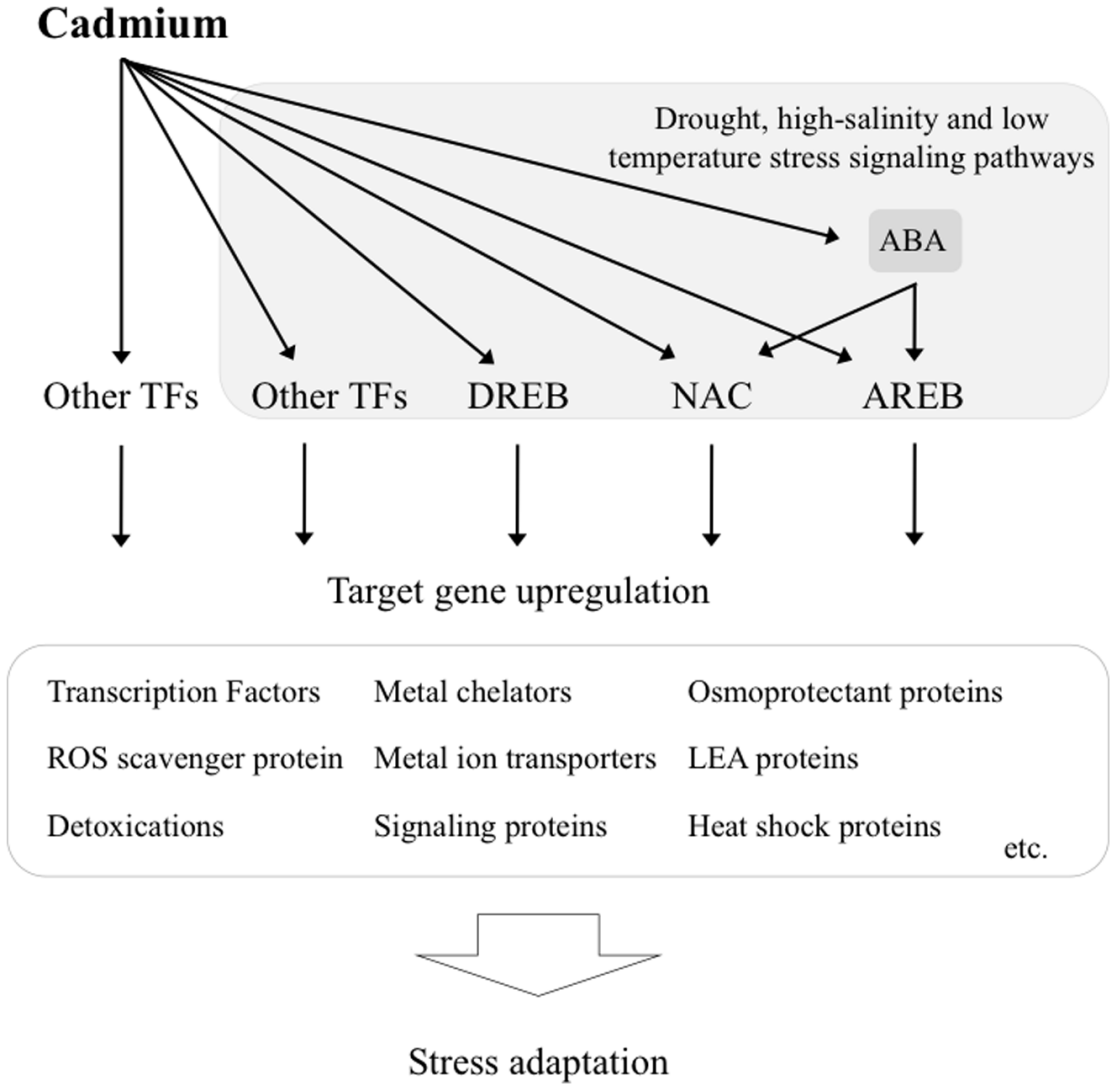
Overview of Cd-dependent signaling cascade that affects drought, high-salinity and low temperature stress signaling pathways. Many abiotic stress-related transcription factors including DREB, bZIP and NAC may function in the signal transduction pathway under Cd exposure. These TFs may regulate the expression of many downstream genes for stress tolerance and responses.

In summary, the sequencing of mRNAs uncovered new genes involved in signal transduction, antioxidation, detoxification and metal transport, and enabled us to develop new hypotheses for the transcriptional network underlying the response to Cd exposure. A highlight was the discovery of a relationship between Cd stress and drought stress. The data suggested that the Cd stress signaling pathway is involved in controlling drought stress signaling pathways that might confer to tolerance to Cd exposure. It is quite possible that the network underlying Cd stress responses and tolerance, which plants have developed to adapt to other stresses, could help to acclimate to Cd exposure because it is a non-essential metal. We have summarized the scheme of signal transduction for acclimation to Cd exposure in rice in Figure 6. As ROS are a toxic byproduct of aerobic metabolism, but also act as signaling molecules in the complex signaling network of cells [5], the generated ROS might act as messengers to activate gene expression in both Cd and abiotic stress pathways. Understanding the relationships between the transduction of different stress signals is useful to develop transgenic rice that show enhanced stress tolerance. Establishing the exact composition and organization of the transcriptional network underlying the response to Cd exposure will provide a robust tool for manipulating the stress tolerance of crops in the future.

## Supporting Information

Figure S1 **Changes in rice morphology after 24 h drought stress.** Rice seedlings grown by hydroponic culture in nutrient media were subjected to drought stress treatment by transferring them to a case without media. The rice seedlings began to show signs of wilting in shoots after 1 h and the changes gradually became more prominent, such that after 24 h of drought stress the shoots were completely wilted compared with control rice not removed from the nutrient media.
(TIFF)

Figure S2 **Identification of GO terms enriched in Cd-responsive transcripts.** Significant GO terms identified by GO enrichment analysis based on the most enriched biological processes associated with variations under Cd exposure are shown in a heatmap of responsive transcripts (left: upregulated transcripts, right: downregulated transcripts). The bar with red-black gradation indicates the level of significance of GO enrichment with the extremes representing statistically significant (red) and non-significant (black) GO terms.
(TIFF)

Figure S3 **qRT-PCR analysis of Cd-responsive genes that may function in drought.** The expression of Cd-responsive drought-related genes was investigated under Cd exposure up to 24 h (black) and drought at 3 h (gray) in qRT-PCR analysis. The x-axis shows treatments and the y-axis shows relative expression. Transcript expression levels were normalized using an internal control (*ubiquitin 1*) and plotted relative to expression in non-treated samples (control) in roots (*R*) and shoots (*S*).
(TIFF)

Figure S4 **Venn diagram analysis of Cd and other stress responsive transcripts.** The resulting four-way Venn diagrams for roots and shoots show the number of transcripts responsive (≥2-fold or ≤ 0.5-fold) to Cd (24 h), drought, high-salinity and low temperature relative to the control (0 d).
(TIFF)

Figure S5 **Expression analysis of gene families that may function in defense against Cd stress.** The graph shows the expression of ROS-scavenging enzyme genes and respiratory burst oxidase homolog (*Rboh*) genes under Cd exposure in RNA-Seq analysis. The x-axis shows genes and y-axis shows relative expression. The black bar shows the relative expression in roots at 1 h, the red bar roots at 24 h, the light blue bar shoots at 1 h and the blue bar shoots at 24 h.
(TIFF)

Figure S6 **Expression patterns of Cd-upregulated antioxidative and detoxification enzymes in various medium conditions by qRT-PCR analysis.** The expression of Cd-upregulated antioxidative and detoxification enzymes was investigated in various liquid media containing different kinds of metal ions by qRT-PCR analysis. The x-axis shows treatments and the y-axis shows relative expression. Transcript expression levels were normalized using an internal control (*ubiquitin 1*) and plotted relative to expression in water (control) at hour 24 in roots (*R*) and shoots (*S*). The transcripts were classified into four groups based on their expression patterns.
(TIFF)

Table S1 **Mapping of RNA-Seq reads obtained from root and shoot samples into the reference IRGSP-1.0 genome sequence.**
(XLS)

Table S2 **Expression (RPKM value) of Ca-responsive transcripts.**
(XLS)

Table S3 **Expression of abiotic stress-related rice genes under Cd exposure.**
(XLS)

Table S4 **PCR primers for qRT-PCR analysis.**
(XLS)

Table S5 **Commonly upregulated transcripts among stresses in roots and shoots.**
(XLS)
