# Supplementary figures and images for: Genome-Wide Transcriptome Analysis Reveals that Cadmium Stress Signaling Controls the Expression of Genes in Drought Stress Signal Pathways in Rice
Source: PLoS One. 2014 May 9;9(5):e96946. doi: 10.1371/journal.pone.0096946 (PMC4016200; doi:10.1371/journal.pone.0096946)

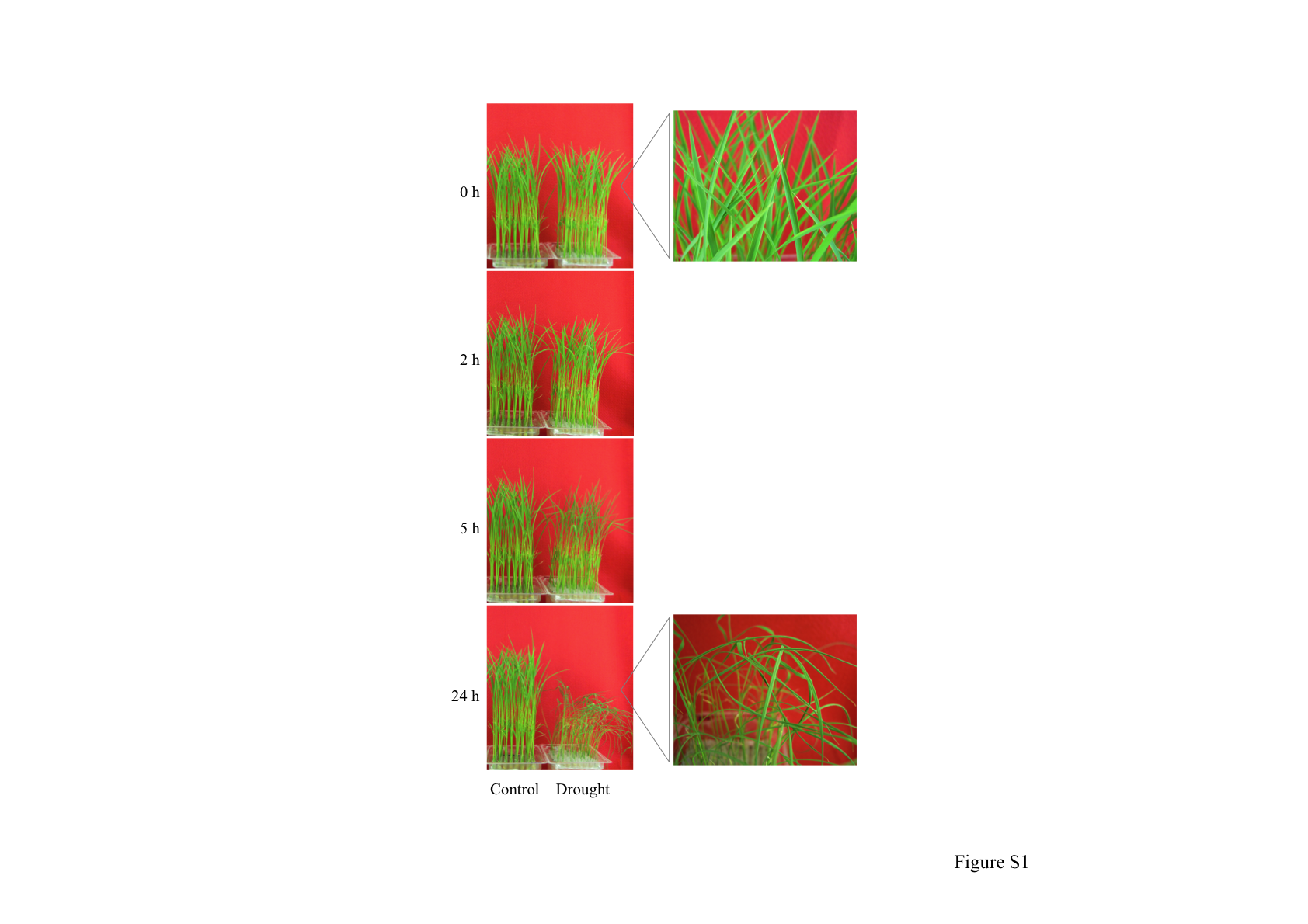

Supplement: Figure S1 — Changes in rice morphology after 24 h drought stress. Rice seedlings grown by hydroponic culture in nutrient media were subjected to drought stress treatment by transferring them to a case without media. The rice seedlings began to show signs of wilting in shoots after 1 h and the changes gradually became more prominent, such that after 24 h of drought stress the shoots were completely wilted compared with control rice not removed from the nutrient media. (TIFF) [file pone.0096946.s001.tiff]

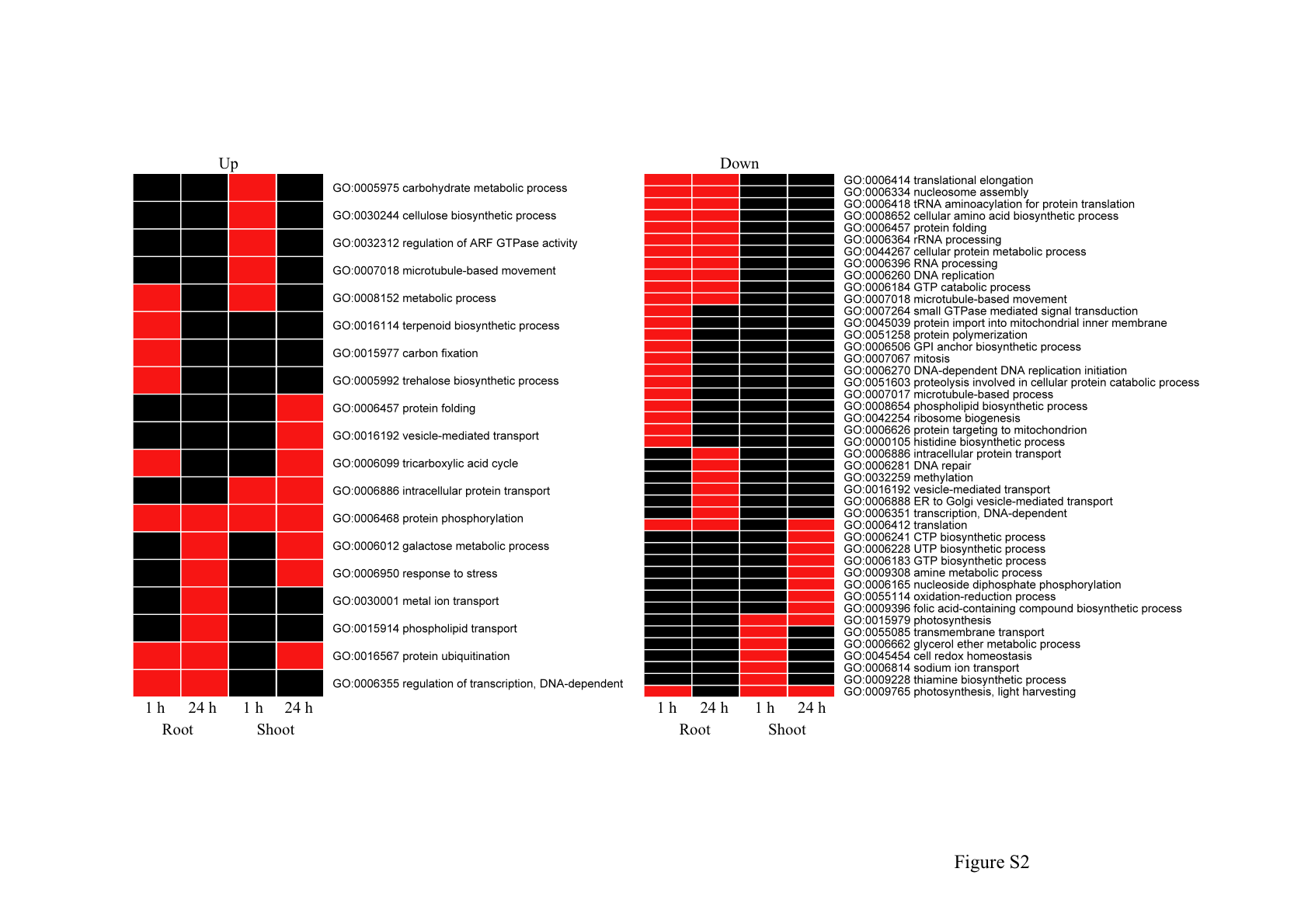

Supplement: Figure S2 — Identification of GO terms enriched in Cd-responsive transcripts. Significant GO terms identified by GO enrichment analysis based on the most enriched biological processes associated with variations under Cd exposure are shown in a heatmap of responsive transcripts (left: upregulated transcripts, right: downregulated transcripts). The bar with red-black gradation indicates the level of significance of GO enrichment with the extremes representing statistically significant (red) and non-significant (black) GO terms. (TIFF) [file pone.0096946.s002.tiff]

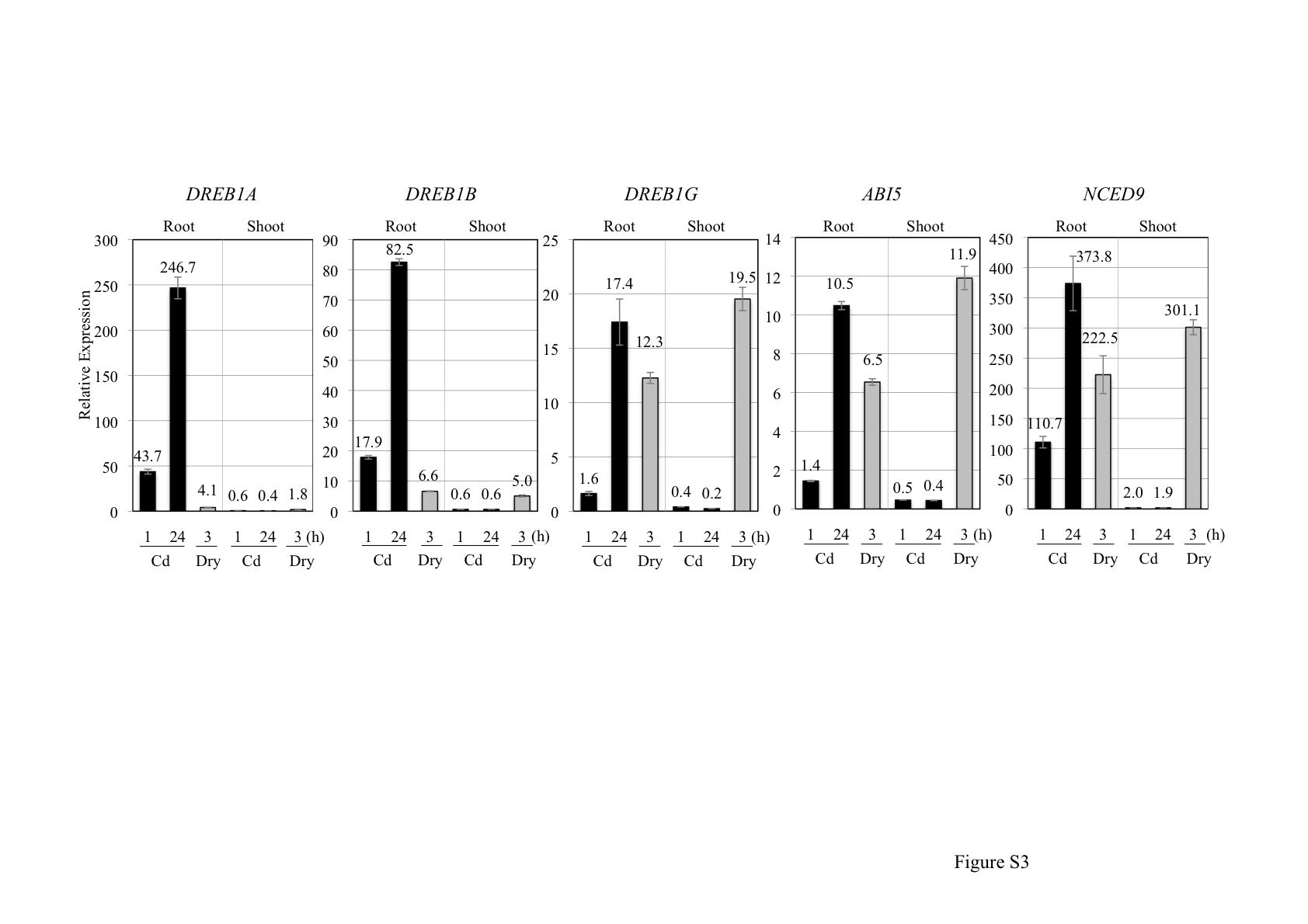

Supplement: Figure S3 — qRT-PCR analysis of Cd-responsive genes that may function in drought. The expression of Cd-responsive drought-related genes was investigated under Cd exposure up to 24 h (black) and drought at 3 h (gray) in qRT-PCR analysis. The x-axis shows treatments and the y-axis shows relative expression. Transcript expression levels were normalized using an internal control (ubiquitin 1) and plotted relative to expression in non-treated samples (control) in roots (R) and shoots (S). (TIFF) [file pone.0096946.s003.tiff]

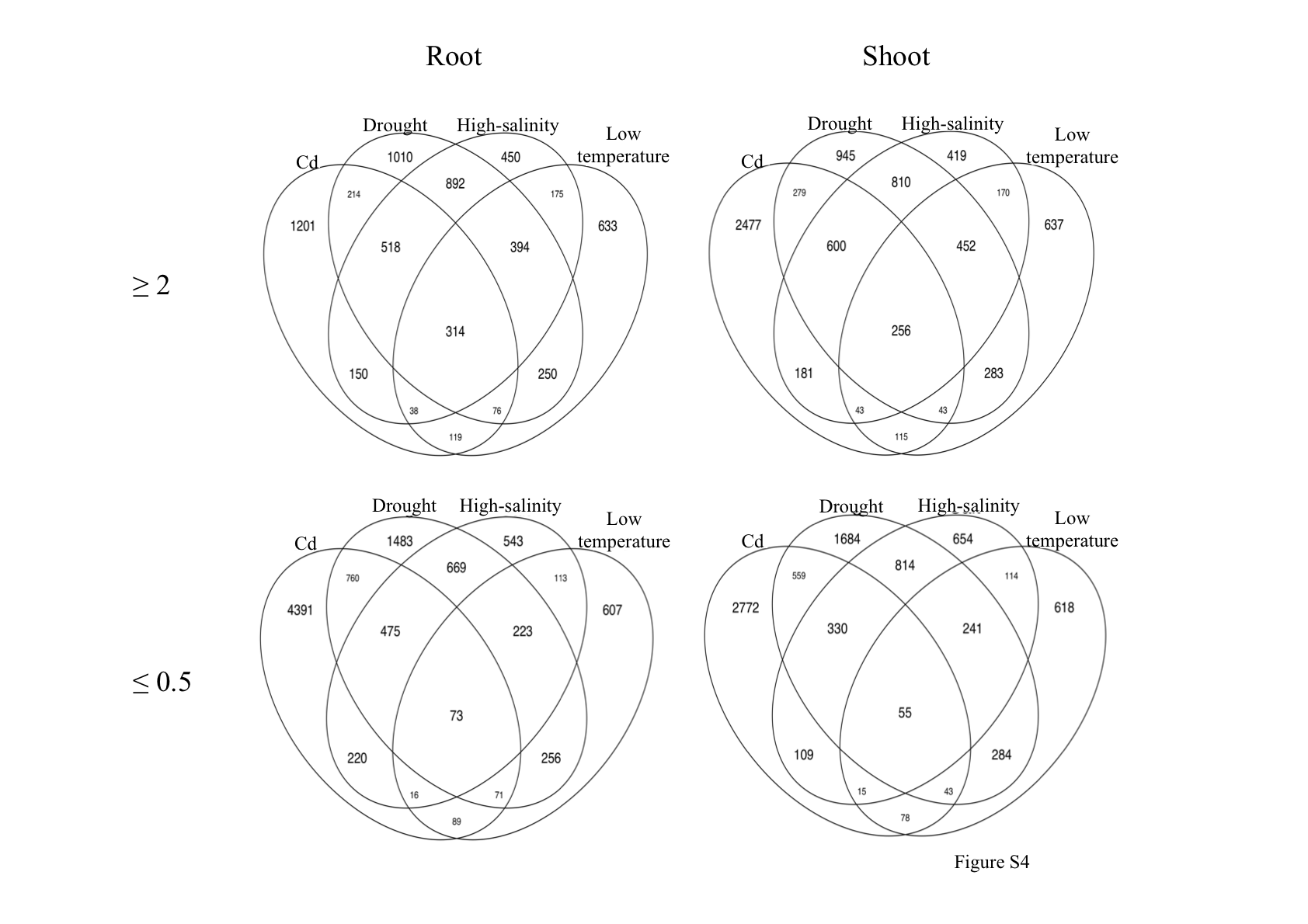

Supplement: Figure S4 — Venn diagram analysis of Cd and other stress responsive transcripts. The resulting four-way Venn diagrams for roots and shoots show the number of transcripts responsive (≥2-fold or ≤ 0.5-fold) to Cd (24 h), drought, high-salinity and low temperature relative to the control (0 d). (TIFF) [file pone.0096946.s004.tiff]

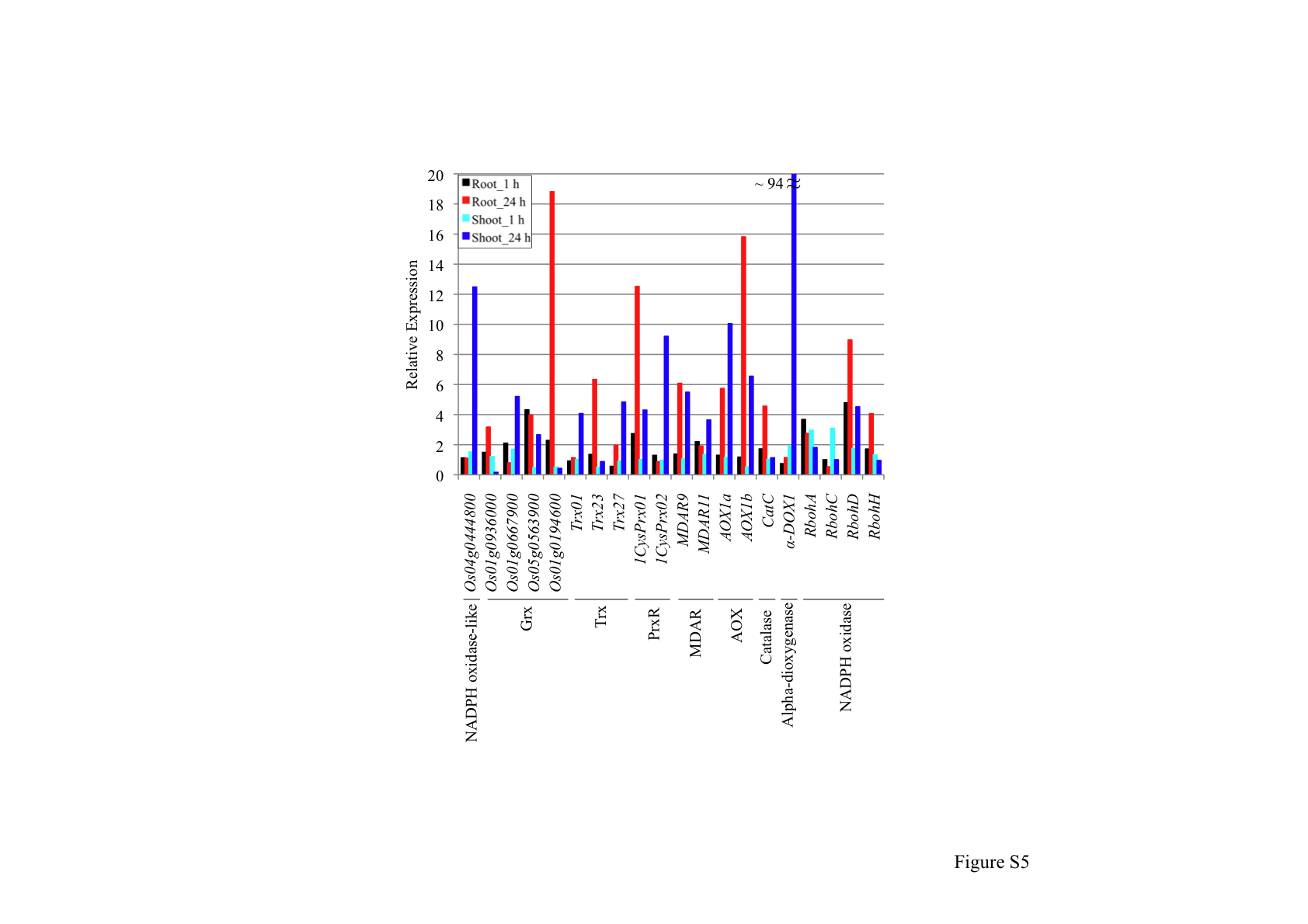

Supplement: Figure S5 — Expression analysis of gene families that may function in defense against Cd stress. The graph shows the expression of ROS-scavenging enzyme genes and respiratory burst oxidase homolog (Rboh) genes under Cd exposure in RNA-Seq analysis. The x-axis shows genes and y-axis shows relative expression. The black bar shows the relative expression in roots at 1 h, the red bar roots at 24 h, the light blue bar shoots at 1 h and the blue bar shoots at 24 h. (TIFF) [file pone.0096946.s005.tiff]

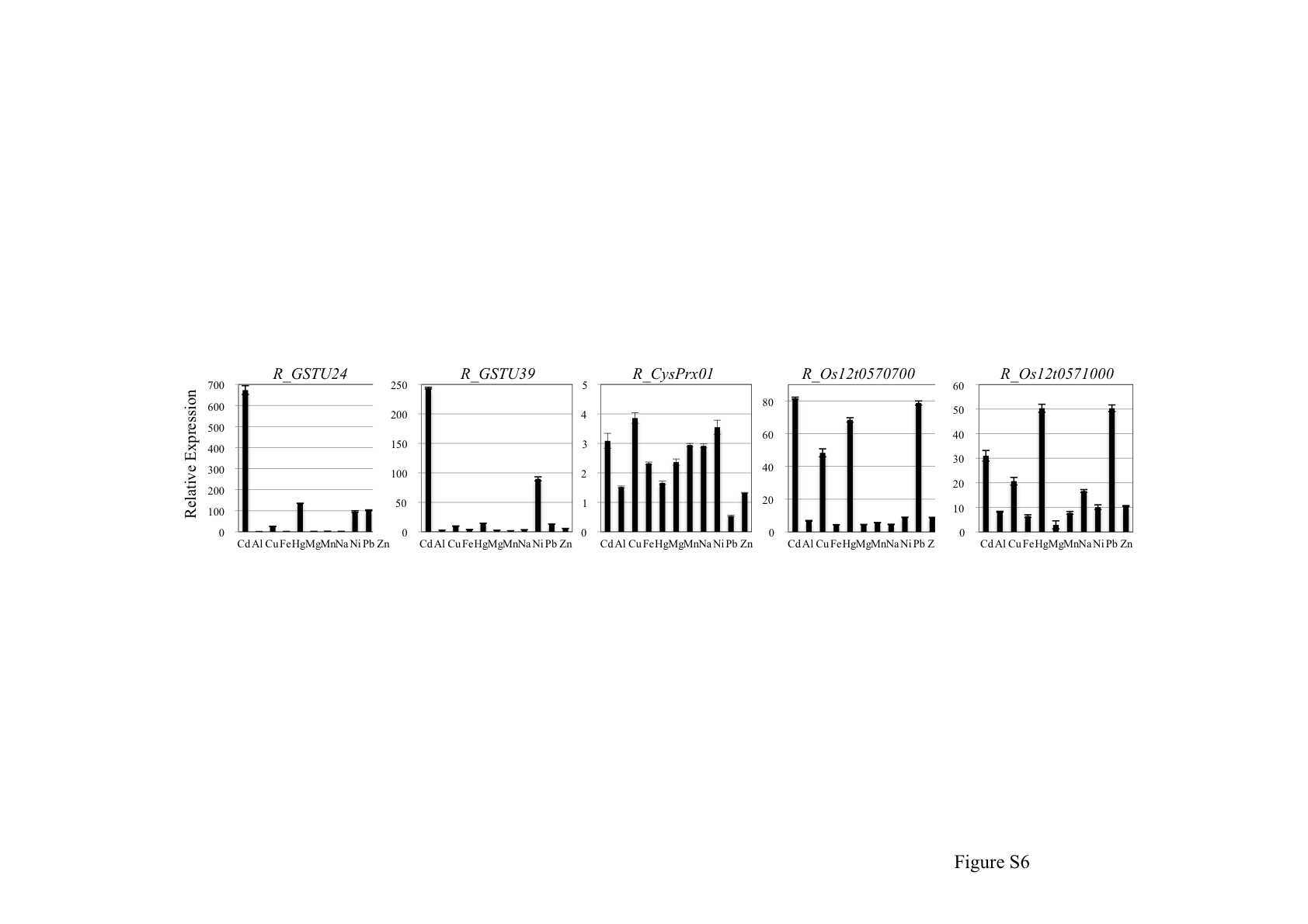

Supplement: Figure S6 — Expression patterns of Cd-upregulated antioxidative and detoxification enzymes in various medium conditions by qRT-PCR analysis. The expression of Cd-upregulated antioxidative and detoxification enzymes was investigated in various liquid media containing different kinds of metal ions by qRT-PCR analysis. The x-axis shows treatments and the y-axis shows relative expression. Transcript expression levels were normalized using an internal control (ubiquitin 1) and plotted relative to expression in water (control) at hour 24 in roots (R) and shoots (S). The transcripts were classified into four groups based on their expression patterns. (TIFF) [file pone.0096946.s006.tiff]
